# EEG-based local brain activity feedback training—tomographic neurofeedback

**DOI:** 10.3389/fnhum.2014.01005

**Published:** 2014-12-12

**Authors:** Herbert Bauer, Avni Pllana

**Affiliations:** Faculty of Psychology, Social, Cognitive and Affective Neuroscience Unit (SCAN-Unit), University of ViennaVienna, Austria

**Keywords:** neurofeedback (NF), sLORETA, tomographic neurofeedback (tNF), EEG-based local brain activity (LBA-) feedback training, rtfMRI neurofeedback

## Abstract

Along with the development of distributed EEG source modeling methods, basic approaches to local brain activity (LBA-) neurofeedback (NF) have been suggested. Meanwhile several attempts using LORETA and sLORETA have been published. This article specifically reports on “EEG-based LBA-feedback training” developed by Bauer et al. (2011). Local brain activity-feedback has the advantage over other sLORETA-based approaches in the way that feedback is exclusively controlled by EEG-generating sources within a selected cortical region of training (ROT): feedback is suspended if there is no source. In this way the influence of sources in the vicinity of the ROT is excluded. First applications have yielded promising results: aiming to enhance activity in left hemispheric linguistic areas, five experimental subjects increased significantly the feedback rate whereas five controls receiving sham feedback did not, both after 13 training runs (U-test, *p* < 0.01). Preliminary results of another study that aims to document effects of LBA-feedback training of the Anterior Cingulate Cortex (ACC) and Dorso-Lateral Prefrontal Cortex (DLPFC) by fMRI revealed more local ACC-activity after successful training (Radke et al., 2014).

## Introduction

Due to the volume conduction genesis of scalp-EEG signals, single- or few-channel EEG-recordings barely convey sufficient information to trace their spatial origin inside the brain. Classical neurofeedback (NF), which typically uses such EEG-recording is, therefore, also spatially unspecific. As a consequence, the “how and where” of changes due to classical NF training in the trainees’ brain is quite uncontrolled and may also vary from person to person, which might reduce the efficacy of NF training.

## Proposed solutions

Concerning improvement of the spatial specificity of NF training, several options have been proposed and partly evaluated during the last two decades. All utilize links between the feedback signal and information from spatially restricted brain areas.

Aiming for high spatial resolution, real time functional magnetic resonance tomography (rtfMRT) was explored by Yoo and Jolesz (2002) and implemented as NF procedures by Posse et al. (2003), Weiskopf et al. (2003) and deCharms et al. (2004). In the ensuing years, the basic usability of rtfMRT-NF was demonstrated in several applications—see Sitaram et al. (2011) and Weiskopf (2012). Near infrared spectroscopy (NIRS), another blood oxygen level dependent (BOLD) technique capable of capturing information on focal cortical activity, has also repeatedly been proposed for brain-computer-interface (BCI) applications and recently utilized in NF procedures (Mihara et al., 2012; Kober et al., 2014). Compared with rtfMRT-NF, NIRS-NF is cost-effective and offers higher portability and usability although it lacks sensitivity to subcortical sources.

In the same period, electromagnetic tomographic techniques have been suggested. LORETA neurofeedback (LNFB)—also referred to as tomographic NF (tNF)—was the first application of this kind, developed by M. Congedo and published in 2004 (Congedo et al., 2004). It is based on LORETA, an inverse solution technique developed by Pascual-Marqui et al. (1994) for localizing sources of multi-channel time or frequency domain EEG/MEG signals within the cortical gray matter volume using a three shell spherical head model. With this procedure the feedback signal is directly linked to the current density (CD) of voxels selected from the solution space as region of training (ROT). Electroencephalogram frequency domain LNFB, as proposed by Congedo et al. (2004), was used, with varying degrees of success in studies by Cannon et al. who investigated its behavioral and cognitive effects and impact on EEG characteristics (Cannon et al., 2006, 2007, 2008, 2009, 2014). Interestingly, the more advanced method “sLORETA”, also developed by Pascual-Marqui (2002) was applied only recently in a tNF-study of 13 children with ADHD by Liechti et al. (2012) (partly also in Maurizio et al., 2014). Aiming to evaluate the therapeutic efficacy of tNF, this study used theta-beta frequency as well as slow cortical potential (SCP) signal components and a single voxel within the anterior cingulate cortex (ACC) as “ROT”. Although no learning in the ACC was observed, this study is quite informative about tNF and more general aspects of NF.

It needs to be emphasized, however, that in all these EEG-based tNF-studies, the spatial specificity of the feedback is still a matter of debate. The (s)LORETA algorithm localizes generating sources by approximating a smooth 3D intra-cortical CD distribution which corresponds to a given EEG/MEG topography. That leads to overlapping current densities from neighboring voxels i.e., to spatial blurring. Consequently, stronger current densities in voxels adjacent to the ROT affect those within the ROT.

An attempt to reduce these consequences has been addressed and discussed by Congedo (2006). In that paper two filters in combination with sLORETA are described and tested on simulated data; one, a spatial filter, reduces the influence of spatial blurring in the ROI, the other filter acts on the input signal, enhancing the signal-to-noise ratio (SNR). However, to our knowledge, no reports on NF-applications of this particular approach have appeared in the literature to date, and Cannon et al. have used the LORETA-based procedure published in 2004 (Congedo et al., 2004) up to 2014. An *a posteriori* elimination of spatial blurring effects by partial correlation analyses on selected ROIs as suggested by Cannon et al. (2009) is inapplicable for controlling the NF learning process directly and also needs to be questioned critically: e.g., correlations in this context are inherently taken as causally determined, which is not assured with LORETA derived CD data sets.

Beamformer spatial filters have evolved with MEG- and BCI-research. A beamformer consists of weights for each electrode with which the scalp signal distribution is spatially filtered to achieve an estimate of the source power at a specific location in the cortex. By constructing beamformers for each location, less blurry 3D estimates of the source power throughout the cerebral cortex can be compiled and generator localization is achievable by identifying local maxima (Van Veen et al., 1997; Green and McDonald, 2009; Grosse-Wentrup et al., 2009).

New approaches for solving the EEG/MEG inverse problem continue to appear in this field e.g., with the aim of identifying and modeling multiple sources of different spatial extent. Haufe et al. (2011), for example, propose a decomposition of the CD into a small number of spatial basis fields; a real-time version, however, is not yet available.

Apart from the spatial blurring issue, it should be borne in mind that only restricted information on the ongoing 3-dimensional neural activity pattern within the cortex is accessible via Scalp potential topographies (SPTs). With respect to this real activity pattern, estimates yield dispersed and overlapping sources in the solution space and it is reasonable to exploit only the local maxima of the estimated activity. Local Brain Activity (LBA-) feedback training was developed with these limitations in mind: EEG topographies are analyzed online by sLORETA and, crucially, feedback is strictly related to generating sources that have their center i.e., local CD maximum, located within the preselected ROT (Bauer et al., 2011).

## The EEG-based LBA-feedback training: the principle

Neurofeedback aims to initiate and maintain instrumental learning. This requires correct and consistent reward during the ongoing training—LBA-feedback enforces that. Scalp potential topographies are generated in most cases by several sources and possible weaker sources within the ROT should not be missed for feedback. Taking these facts into account “simultaneous multiple sources (SMS-) LORETA” was developed as the core procedure of LBA-feedback. It identifies all generator loci i.e., all local maxima, in sLORETA-derived CD solutions automatically and rapidly utilizing individual electrode coordinates projected on a 3-shell realistic head model (Pllana and Bauer, 2008, 2011).

The time-domain SMS-LORETA procedure consists of

1. an iteration loop: recorded potential topography > *sLORETA transformation > storage of the maximum current density’s spatial location > calculation of a forward solution (i.e., surface potential topography) that corresponds to a standardized source at this location > cumulative subtraction of this forward solution from the recorded potential topography > as new input to sLORETA until the initially recorded potential topography is flat*; and
2. a “spatial” cluster analysis of all stored maximum CD locations; and
3. the identification of all cluster centers which then are taken as loci of generating sources with the maximum CD within each cluster as their corresponding strength—for details see Pllana and Bauer (2008, 2011).

Screening applications of quasi continuous LBA-feedback revealed quite infrequent feedback with sometimes long waiting epochs and turned out to be insufficient to initiate learning. These observations have led to the current implementation of LBA-feedback training which is executed in a stepwise task-/stimulus-linked manner. This strategy also has the advantage that it allows SNR-enhancement by application of single-trial evoked potential (EP) estimation. Trainees are presented with short duration stimuli or tasks (1–8 s) via computer display. To a greater or lesser extent these involve the ROT-structures. Trainees are asked to respond to these stimuli/tasks accordingly, and mentally retain these responses during the presentation period. EOG- and pre-stimulus-baseline corrected SPTs are extracted from the ongoing multi-channel EEG at selectable latencies and SMS-LORETA analyzed. If this analysis identifies a source within the predefined ROT its strength determines the brightness of a green feedback signal presented as a narrow frame around the stimulus/task presentation area. If no source is detected within the ROT the narrow frame remains or turns gray. This feedback is updated after each stimulus/task according to the current SMS-LORETA result. As a crucial additional instruction, trainees are asked to try to keep this frame green as long and as bright as possible.

## First applications

In order to explore the feasibility of LBA-feedback, a screening study was performed that investigated if subjects are able to learn to enhance the activity within left hemispheric linguistic areas (BA 6,21,22,40,44,45) by means of the task-linked procedure (Bauer et al., 2011). Ten healthy right-handed subjects participated in daily training sessions on seven consecutive working days beginning on Mondays. Five subjects received consistent feedback (experimental group; EG) the other five sham feedback (control group; CG). A session had 2 runs of 120 item presentations each. Items were sketches of simple actions, each presented on a computer screen for 3 s with varying inter-stimulus intervals of 6 +/− 2 s. The subjects’ task was to covertly name the verb that corresponded to the presented item and, simultaneously, turn the gray frame around the item presentation area as intensively green as possible as the feedback signal. While each item was presented, 59-channel DC-EEG signal epochs (equidistant montage, 125 samples/s, corrected for eye movement artifacts, referenced to a 500 ms pre-stimulus baseline) were recorded and immediately analyzed by SMS-LORETA at three latency windows. Members of the EG received feedback via green frames whenever generating sources were detected within the ROT. The intensity of the green was proportional to the sum of the strengths of the identified sources. Green feedback for members of the CG was randomly presented with varying intensity in 20% of the items, which corresponded to the average initial feedback rate of the EG. The second run of the last session was a so-called “transfer run” i.e., no feedback was shown, but subjects were informed about this and instructed to behave as they did during the more recent runs. The aim of this study was to check whether trainees who receive correct feedback are able to increase the feedback rate across runs where in controls this rate does not change. Taking the relative feedback rate per run as a measure of the NF learning process, we observed an increase in the EG across the runs but no change or even a decrease in the CG. The feedback rate increase i.e., the feedback rate difference between the transfer and the initial run, was significantly higher in the EG than in the CG (Mann-Whitney U test; *p* < 0.01).

**Figure 1:**
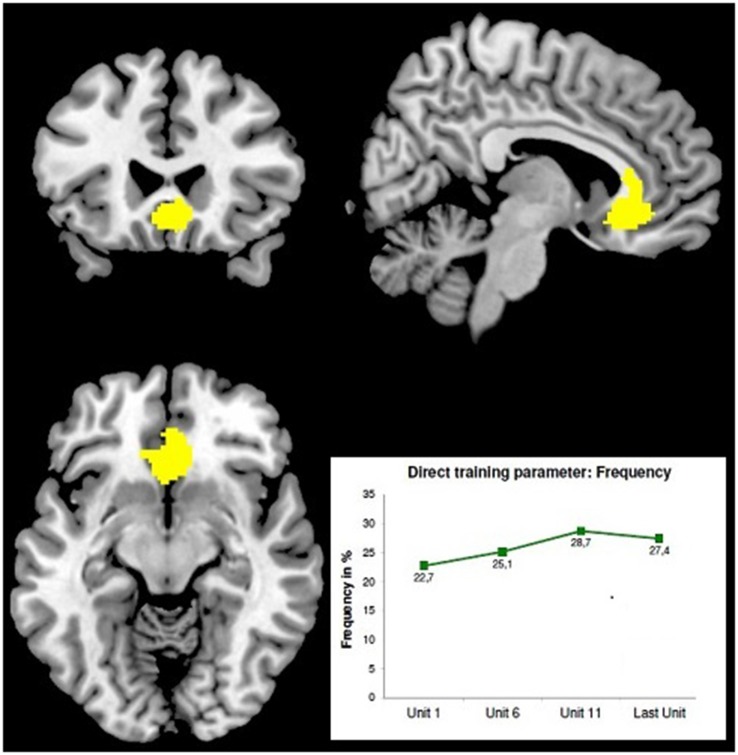
**Stronger BOLD-effect with the Age-Stroop after LBA-feedback training of the ACC [maximum at 6 24 −10; *z* = 7.77; FMRI-image thresholded at *T* = 4.86; MRT: 3T, TE = 28, TR = 2 s, 34 slices, 3.3 mm^3^ voxel size] adapted from Radke et al. (2014)**. The minor displacement of the BOLD-maximum from the ROT may result from slightly inaccurate individual ROT-localization—IHMs were already used, but no localizer EPs. Bottom right: averaged learning curve of 10 subjects.

First very preliminary results of a recent screening study performed by B. Derntl’s group (RWTH Aachen, Germany) demonstrate the effect of LBA-feedback training on the behavioral and neurophysiological level (Radke et al., 2014). Ten right-handed subjects were asked to enhance the activity in their ACC (ROT: BA24/32) and another 10 subjects to enhance the activity in their Dorso-Lateral Prefrontal Cortex (DLPFC) (ROT:BA46). The NF-training consisted of 10 sessions with two consecutive runs per day, each consisting of 70 stimuli of a Stroop-test variant, the “Age-Stroop”. The “Age-Stroop” items were portraits of people of a range of ages, annotated congruently or incongruently (50/50%) as “YOUNGER/MIDDLE/OLDER” and were presented for 3 s in inter-stimulus intervals of 4 +/− 1 s. Trainees had to judge the person‘s age as younger, middle or older by button press. While each item was presented, 58-channel DC-EEG epochs (equidistant montage, 125 s/s, corrected for eye movement artifacts and a 500 ms pre-stimulus baseline) were recorded and immediately analyzed by SMS-LORETA at three latency windows. In order to improve localization accuracy, individual head models (IHMs) were used. Whenever a generating source occurred in the ROT the gray feedback-frame turned green as a feedback signal, whereas its intensity corresponded to the strength of the detected source. This feedback was updated after each item according to the current outcome. The subjects were instructed to keep this frame green for as long and as intensely as possible. First results from the ACC-group (*N* = 10) showed a significant increase of the mean feedback-frequency during training (*p* < 0.05). Functional magnetic resonance tomography checks with this group before and after the training using an event-related design and separate sequences of Age-Stroop items and Emotional-Stroop items [portraits of fearful, happy and sad faces, annotated congruently or incongruently (50/50%) as “FEARFUL/HAPPY/SAD”; portrayed emotions had to be judged] yielded the following preliminary observations: (1) reaction times to Emotional-Stroop items were longer after the feedback training; and (2) a voxel-cluster in the mid-orbital gyrus extending to the ACC showed more activity with the Age-Stroop after than before the training—see Figure 1.

## Discussion

Although results only exist for these two preliminary studies to date, it seems clear that EEG-based feedback training of LBA is feasible. As the first study demonstrates this for a rather large ROT, the latter confirms it for a quite small area and moreover on a neurophysiological level using fMRI.

Most NF control studies focus on questions such as “does it work” or “how well does it work”, but only a few are concerned with the changes in the trainees’ brain specifically caused by NF. Therefore, also the specificity and efficacy of a particular NF protocol are predominantly determined by its therapeutic outcome described on the behavioral and introspective level, not by specific changes in particular brain structures—the “where and how it works” was rarely addressed. How inadequate such efficacy measures can be became apparent in the Liechti et al. (2012) study: unsuccessful tNF training (feedback of single voxel current densities) of ACC activity in children with ADHD was, nevertheless, accompanied by significant clinical improvement. Not control over ACC activity was efficient, but the training process on its own appeared to be an effective behavioral and cognitive treatment at least for ADHD patients. More generally, in case a consistent localizable and NF-mediated change in brain activity can be observed, we can assume that the clinical or behavioral NF outcome may be due to this change. Validation using fMRI is possible directly with SCP-NF as aimed at by Hinterberger et al. (2003). With frequency domain NF, fMRI checks allow only indirect proofs (e.g., Kinreich et al., 2012). In principle, however, such validations are essential in order to evaluate the extent to which NF acts directly on the neurophysiological level.

Compared with fMRI, sLORETA has its limits: (1) not all activity hot spots within the cerebral cortex at a particular time can be detected; (2) the spatial resolution is predominantly a matter of implementation and varies between 5–8 mm and; (3) the accuracy of localization depends on the source configuration, the precision of the head model used and the adequate capturing of the SPTs.

Concerning point (1) there is no room for improvement. As already been mentioned, full information on the 3-dimensional intra-cortical activity pattern is not accessible via 2-dimensional SPTs. Similarly, point (2)—resolution—can hardly be improved because sLORETA yields smooth solutions with attainment and optimization of source localization.

Accuracy (point 3), however, is improvable since the head model can be made more realistic and a sufficient number of electrodes can be applied. Therefore the LBA-feedback procedure uses already IHMs and session-specific Cartesian coordinates of 58 electrodes.

Individual head models in current use are constructed by reshaping all parts of the standard BEM-based 3-shell head model including the Brodmann area (BA) map according to a trainee’s individual electrode coordinates. Afterwards, the solution space within the newly shaped cerebral volume is readjusted and the appropriate lead field matrix recalculated. In this way, individual voxel-electrode distances are taken into account.

Utilizing localizer procedures to exactly locate ROTs can additionally improve the spatial accuracy of LBA-feedback training. For many cortical structures i.e., possible targets for LBA-feedback training, characteristic EP components are known which are generated in these structures, e.g., aSSR for A1 or ERN and FRN for the ACC. Pre-training EP acquisition and subsequent sLORETA source localization of the appropriate EP-components can indicate the center location for individual ROT definition. More generally, acquisition of several localizer EPs together, e.g., from V1, A1, ACC and DLPFC, may even enable realignment of BA maps within IHMs.

Since SPTs are the only source of information in this analysis it is extremely important to capture them accurately. Applying electrodes on the scalp means sampling in space, where the same regularities must be observed as in the time domain, but for spatial frequencies i.e., potential changes over distances. Studies have shown that about 60 electrodes equally distributed over the scalp are sufficient to avoid spatial aliasing (Srinivasan et al., 1998; Luu et al., 2001; Freeman et al., 2003). The drawback of spatial under-sampling is twofold: (a) higher spatial frequencies remain undetected; and (b) unidentifiable spatial aliasing frequencies will be generated, which cannot be filtered out.

Altogether, the application of IHMs, session-specific electrode coordinates and appropriate localizer methods makes EEG-based LBA- and rtfMRI-feedback comparable, as far as spatial accuracy is concerned.

With NF applications, in general, deactivation of cortical structures is also of interest. However, EEG-based LBA-feedback as described above needs to be explored and evaluated in this respect—because initiating and pursuing NF-learning of targeted deactivation via inverse solutions based on time domain EEG signals is presumably more complex than learning of targeted activation. Since classical NF is dominated by using EEG frequency components/bands, frequency-domain LBA-feedback preferably is intended to become implemented. This way also targeted deactivation at least for some structures is achievable, for example, by enhancing local alpha activity.

## Conclusion

With the development and publication of the EEG-based LBA-feedback procedure, real EEG-based local/targeted brain activity feedback training is available for the first time. Utilizing knowledge on the functional role of cortical structures and neuronal networks gathered by social, cognitive and affective neuroscience, this procedure is particularly suited to enable NF with enhanced physiological specificity. EEG-based LBA-feedback enables various *targeted* NF applications: in Neurology and neurological rehabilitation, as psychiatric/psychological treatments and as training to expand cognitive and behavioral abilities of healthy humans. In order to fine-tune all constituents of tNF, however, further intensive research is necessary.

## Conflict of interest statement

The authors declare that the research was conducted in the absence of any commercial or financial relationships that could be construed as a potential conflict of interest.

## References

[B1] Bauer H. Pllana A. Sailer U. (2011). The EEG-based local brain activity (LBA-) feedback training. Act. Nerv. Super Rediviva 53, 107–113.

[B2] Cannon R. L. Baldwin D. R. Diloreto D. J. Phillips S. T. Shaw T. L. Levy J. J. (2014). LORETA neurofeedback in the precuneus: operant conditioning in basic mechanisms of self-regulation. Clin. EEG Neurosci. 45, 238–248. 10.1177/1550059413512796 24590872

[B3] Cannon R. Congedo M. Lubar J. F. Hutchens T. (2009). Differentiating a network of executive attention: LORETA neurofeedback in anterior cingulate and dorsolateral prefrontal cortices. Int. J. Neurosci. 119, 404–441. 10.1080/00207450802480325 19116846

[B4] Cannon R. Lubar J. F. Congedo M. Thornton K. Towler K. Hutchens T. (2007). The effect of neurofeedback training in the cognitive division of the anterior cingulate gyrus. Int. J. Neurosci. 117, 337–357. 10.1080/00207450500514003 17365119

[B5] Cannon R. Lubar J. F. Gerke A. Thornton K. Hutchens T. McCammon V. (2006). EEG spectral-power and coherence: LORETA neurofeedback training in the anterior cingulate gyrus. J. Neurother. 10, 5–31 10.1300/j184v10n01_02

[B6] Cannon R. Lubar J. F. Sokhadze E. Baldwin D. R. (2008). LORETA neurofeedback for addiction and the possible neurophysiology of psychological processes influenced: a case study and region of interest analysis of LORETA neurofeedback in right anterior cingulate cortex. J. Neurother. 12, 227–241 10.1080/10874200802501948

[B7] Congedo M. (2006). Subspace projection filters for real-time brain electromagnetic imaging. IEEE Trans. Biomed. Eng. 53, 1624–1634. 10.1109/tbme.2006.878055 16916097

[B8] Congedo M. Lubar J. F. Joffe D. (2004). Low-resolution electromagnetic tomography neurofeedback. IEEE Trans. Neural Syst. Rehabil. Eng. 12, 387–397. 10.1109/tnsre.2004.840492 15614994

[B9] deCharms R. C. Christoff K. Glover G. H. Pauly J. M. Whitfield S. Gabrieli J. D. (2004). Learned regulation of spatially localized brain activation using real-time fMRI. Neuroimage 21, 436–443. 10.1016/j.neuroimage.2003.08.041 14741680

[B10] Freeman W. J. Holmes M. D. Burke B. C. Vanhatalo S. (2003). Spatial spectra of scalp EEG and EMG from awake humans. Clin. Neurophysiol. 114, 1053–1068. 10.1016/s1388-2457(03)00045-2 12804674

[B11] Green J. J. McDonald J. J. (2009). “A practical guide to beamformer source reconstruction for EEG,” in Brain Signal Analysis: Advances in Neuroelectric and Neuromagnetic Methods, ed Handy T. C. (Cambridge, MA: The MIT Press), 79–98.

[B12] Grosse-Wentrup M. Liefhold C. Gramann K. Buss M. (2009). Beamforming in noninvasive brain-computer interfaces. IEEE Trans. Biomed. Eng. 56, 1209–1219. 10.1109/tbme.2008.2009768 19423426

[B13] Haufe S. Tomioka R. Dickhaus T. Sannelli C. Blankertz B. Nolte G.. (2011). Large-scale EEG/MEG source localization with spatial flexibility. Neuroimage 54, 851–859. 10.1016/j.neuroimage.2010.09.003 20832477

[B14] Hinterberger T. Veit R. Strehl U. Trevorrow T. Erb M. Kotchoubey B.. (2003). Brain areas activated in fMRI during self-regulation of slow cortical potentials (SCPs). Exp. Brain Res. 152, 113–122. 10.1007/s00221-003-1515-4 12830347

[B15] Kinreich S. Podlipsky I. Intrator N. Hendler T. (2012). Categorized EEG neurofeedback performance unveils simultaneous fmri deep brain activation. Mach. Learn. Interpretation Neuroimaging Lect. Notes Comput. Sci. 7263, 108–115 10.1007/978-3-642-34713-9_14

[B16] Kober S. E. Wood G. Kurzmann J. Friedrich E. V. C. Stangl M. Wippel T.. (2014). Near-infrared spectroscopy based neurofeedback training increases specific motor imagery related cortical activation compared to sham feedback. Biol. Psychol. 95, 21–30. 10.1016/j.biopsycho.2013.05.005 23714227

[B17] Liechti M. D. Maurizio S. Heinrich H. Jäncke L. Meier L. Steinhausen H.-C.. (2012). First clinical trial of tomographic neurofeedback in attention-deficit/hyperactivity disorder: evaluation of voluntary cortical control. Clin. Neurophysiol. 123, 1989–2005. 10.1016/j.clinph.2012.03.016 22608481

[B18] Luu P. Tucker D. M. Englander R. Lockfeld A. Lutsep H. Oken B. (2001). Localizing acute stroke-related EEG changes: assessing the effects of spatial undersampling. J. Clin. Neurophysiol. 18, 302–317. 10.1097/00004691-200107000-00002 11673696

[B19] Maurizio S. Liechti M. D. Heinrich H. Jäncke L. Steinhausen H. C. Walitza S.. (2014). Comparing tomographic EEG neurofeedback and EMG biofeedback in children with attention-deficit/hyperactivity disorder. Biol. Psychol. 95, 31–44. 10.1016/j.biopsycho.2013.10.008 24211870

[B20] Mihara M. Miyai I. Hattori N. Hatakenaka M. Yagura H. Kawano T.. (2012). Neurofeedback using real-time near-infrared spectroscopy enhances motor imagery related cortical activation. PLoS One 7:e32234. 10.1371/journal.pone.0032234 22396753 PMC3292558

[B21] Pascual-Marqui R. D. (2002). Standardized low resolution brain electromagnetic tomography (sLORETA): technical details. Methods Find Exp. Clin. Pharmacol. 24D, 5–12. 12575463

[B22] Pascual-Marqui R. D. Michel C. M. Lehmann D. (1994). Low resolution electromagnetic tomography: a new method for localizing electrical activity in the brain. Int. J. Psychophysiol. 18, 49–65. 10.1016/0167-8760(84)90014-x 7876038

[B23] Pllana A. Bauer H. (2008). Localization of simultaneous multiple sources using SMS-LORETA. arXiv: 2008; 0806.4845 [q-bio], Available online at: http://arxiv.org/ftp/arxiv/papers/0806/0806.4845.pdf

[B24] Pllana A. Bauer H. (2011). BEM-based SMS-LORETA - an advanced method to localize multiple simultaneously active sources in the cerebral cortex. arXiv: 2011; Available online at: http://arxiv.org/ftp/arxiv/papers/1106/1106.2679.pdf

[B25] Posse S. Fitzgerald D. Gao K. Habel U. Rosenberg D. Moore G. J.. (2003). Real-time fMRI of temporolimbic regions detects amygdala activation during single-trial self-induced sadness. Neuroimage 18, 760–768. 10.1016/s1053-8119(03)00004-1 12667853

[B26] Radke S. Kellermann T. Kogler L. Schuch S. Bauer H. Derntl B. (2014). Training the ACC with localized EEG-neurofeedback - a pioneer study. Poster presented at the 2nd Conference of the European Society for Cognitive and Affective Neuroscience (ESCAN) Dortmund, Germany.

[B28] Sitaram R. Lee S. Ruiz S. Birbaumer N. (2011). “Real-time regulation and detection of brain states from fMRI signals,” in Neurofeedback and Neuromodulation Techniques and Applications, eds Coben R. Evans J. R. (New York: Academic press), 227–249.

[B27] Srinivasan R. Tucker D. M. Murias M. (1998). Estimating the spatial Nyquist of the human EEG. Behav. Res. Methods Instrum. Comput. 30, 8–19 10.3758/bf03209412

[B29] Van Veen B. D. Van Drongelen W. Yuchtman M. Suzuki A. (1997). Localization of brain electrical activity via linearly constrained minimum variance spatial filtering. IEEE Trans. Biomed. Eng. 44, 867–880. 10.1109/10.623056 9282479

[B30] Weiskopf N. (2012). Real-time fMRI and its application to neurofeedback. Neuroimage 62, 682–692. 10.1016/j.neuroimage.2011.10.009 22019880

[B31] Weiskopf N. Veit R. Erb M. Mathiak K. Grodd W. Goebel R.. (2003). Physiological self-regulation of regional brain activity using real-time functional magnetic resonance imaging (fMRI): methodology and exemplary data. Neuroimage 19, 577–586. 10.1016/s1053-8119(03)00145-9 12880789

[B32] Yoo S. S. Jolesz F. A. (2002). Functional MRI for neurofeedback: feasibility study on a hand motor task. Neuroreport 13, 1377–1381. 10.1097/00001756-200208070-00005 12167756

